# Kissing Balloon Dilatation of a Transcatheter Mitral Valve Replacement Within an Extra-Large D-Shaped Annuloplasty Ring

**DOI:** 10.1016/j.jaccas.2025.103279

**Published:** 2025-03-12

**Authors:** Harish Sharma, Mohsin Z.S. Ullah, Sagar N. Doshi, M. Adnan Nadir

**Affiliations:** a Department of Cardiology, Queen Elizabeth Hospital Birmingham, Birmingham, United Kingdom; b Institute of Cardiovascular Sciences, University of Birmingham, Birmingham, United Kingdom

**Keywords:** leaflet modification, paravalvular leak, transcatheter mitral valve replacement, valve-in-ring

## Abstract

Paravalvular leak (PVL) is a recognized complication of transcatheter mitral valve replacement when a circular transcatheter heart valve (THV) is implanted within a D-shaped annuloplasty ring. Here we present the use of a novel kissing balloon dilatation method to improve THV apposition within the annuloplasty ring and reduce PVL.

## Visual Summary

**Figure:**
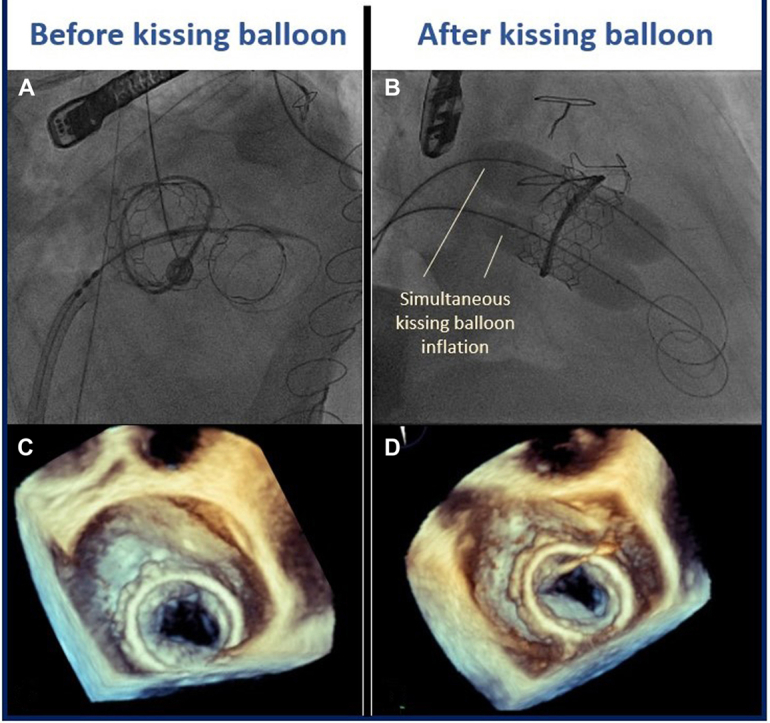

Valve-in-ring transcatheter mitral valve replacement (TMVR) has emerged as a treatment option for patients with recurrence of mitral regurgitation (MR) after previous mitral annuloplasty who are at prohibitive risk of re-do surgery.^1^ In the current absence of purpose-built fully percutaneous valves that can be implanted in an annuloplasty ring, TMVR currently involves the implantation of a circular transcatheter heart valve (THV) designed for transcatheter aortic valve replacement (TAVR). However, because the mitral annuloplasty ring is D shaped, this often results in a paravalvular intra-ring leak, particularly with extra-large rings (>36 mm) which until now, have been thought to be unsuitable for valve-in-ring (ViR) TMVR.

**Take-Home Messages**

• Transcatheter mitral valve replacement can be successfully performed within a 36-mm annuloplasty ring.
• Post-dilatation of the valve is necessary to reduce the extent of paravalvular leak.
• Using a kissing balloon inflation over 2 parallel wires can improve conformity of a circular transcatheter heart valve with a D-shaped ring.
• Residual paravalvular leak may need to be addressed with a vascular plug. This can be performed in conjunction with the kissing balloon inflation technique.

Annuloplasty rings of >34 mm diameter are frequently used to treat degenerative MR^2^ owing to annular dilation and because larger rings can help minimize systolic anterior motion of the anterior mitral valve leaflet (AMVL) in patients with excessive leaflet tissue.^3^ However, recurrence of MR in such patients poses a treatment dilemma as redo surgery carries significant risk. Transcatheter treatment by TMVR is also challenging because of: 1) the significantly larger internal area of the ring (704 mm^2^) relative to the largest widely available balloon-expandable THV (660 mm^2^); 2) the difference in shape between the D-shaped ring the circular THV; and 3) the risk of potential obstruction of the neo–left ventricular outflow tract (LVOT) after valve-in-ring TMVR from excessive leaflet tissue.

The risk of risk of neo-LVOT obstruction can be mitigated with the use of leaflet modification by laceration of the anterior mitral leaflet to prevent outflow obstruction (LAMPOON),^4^, ^5^, ^6^, ^7^ but there are few strategies to address the malapposition of the THV within the ring.

We describe here a case of successful ViR TMVR within a 36-mm annuloplasty ring, facilitated by means of tip-to-base LAMPOON. After identification of residual paravalvular leak (PVL) with hemolysis, we describe the use of a novel kissing balloon dilatation to modify the THV to an elliptical shape to better fit the annuloplasty ring, combined with closure of the PVL by implantation of a vascular plug.

## History of Presentation

A 75-year-old man with a history of mitral valve surgery in 2015 for AMVL prolapse and moderate (bicuspid) aortic regurgitation with left ventricular dilation presented with recently developed progressive dyspnea. He remained well for a number of years after his surgery before the current presentation.

## Past Medical History

The mitral valve was repaired with the use of a 36-mm Physio II annuloplasty ring (Edwards Lifesciences) with NeoChord implantation, and an aortic valve replacement (AVR) was performed with a bioprosthetic Perimount Magna Ease valve (Edwards Lifesciences).

Other comorbidities included chronic kidney disease (stage 3a), pectus excavatum, low body weight, and chronic anemia.

## Investigations

**Figure 1:**
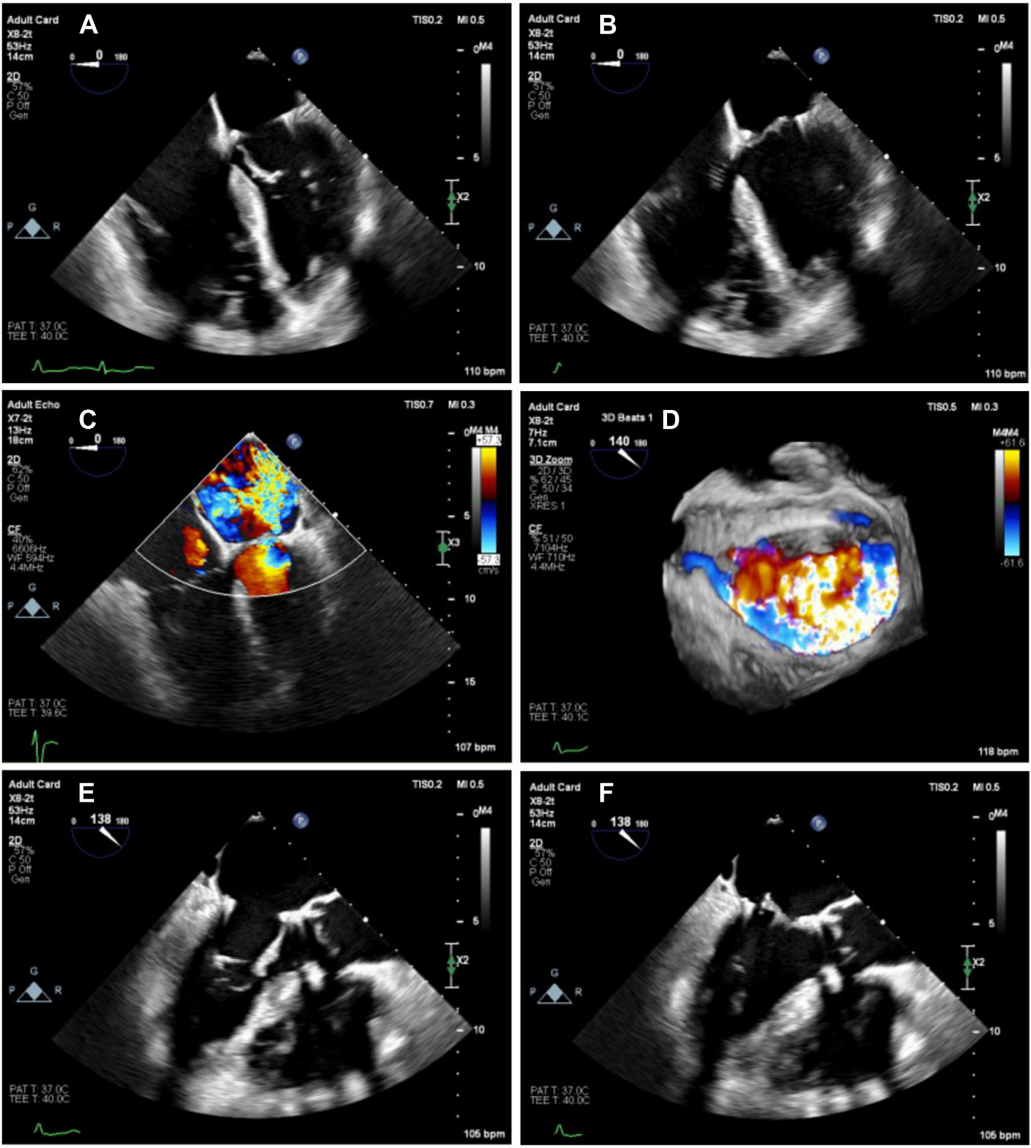
Transesophageal Echocardiography Opening (A and E) and closing (B and F) of the mitral valve in the 4-chamber and left ventricular outflow tract views, respectively. (C and D) The posteriorly directed jet of severe mitral regurgitation in the 4-chamber and 3-dimensional en-face views, respectively.

Transesophageal echocardiography (TEE) revealed a well-functioning AVR, however there was severe MR due to AMVL prolapse and redundant chordal tissue (Figure 1). There was also evidence of severe pulmonary hypertension with impaired right ventricular systolic function. The left ventricle was nondilated with preserved systolic function. Computed tomography (CT) demonstrated neo-LVOT area of 200 mm^2^. A significant reduction in LVOT area and an elongated untethered anterior mitral leaflet increased the risk of LVOT obstruction.

## Management

Diagnosis of severe symptomatic MR was made. The patient had a number of comorbidities rendering repeated surgical intervention high risk. His case was discussed by the heart team and it was felt that the MR should be managed percutaneously. Transcatheter edge-to-edge repair (TEER) was not feasible owing to almost complete absence of posterior leaflet. Valve-in-ring TMVR was therefore considered with the use of a Sapien S3 Ultra (Edwards Lifesciences) THV. Because of the risk of LVOT obstruction in the neo-LVOT, the TMVR was planned with the use of LAMPOON.

**Figure 2:**
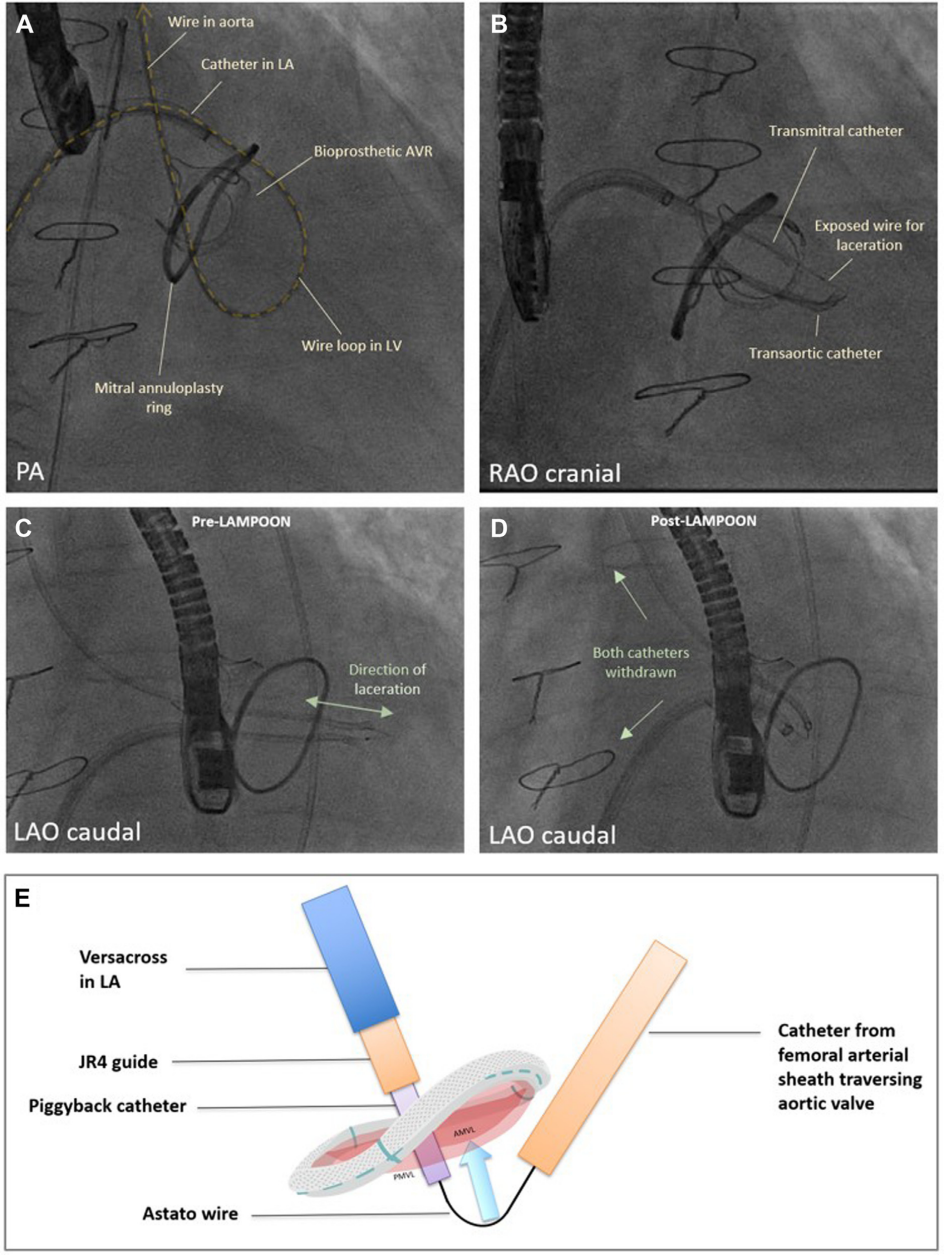
Fluoroscopic Images of the LAMPOON Procedure (A) Initially a transmitral wire is crossed into the left ventricle and snared from a transaortic catheter and then externalized. The 2 insulating guiding catheters are then withdrawn to expose a section of wire, (B) which is positioned in the center of the leaflet to be lacerated. (C and D) The wire is electrified and withdrawn by drawing back the transmitral and transaortic catheters simultaneously. (E) Illustration of the LAMPOON procedure. LA = left atrium; LAO = left anterior oblique; PA = postero-anterior; RAO = right anterior oblique.

**Figure 3:**
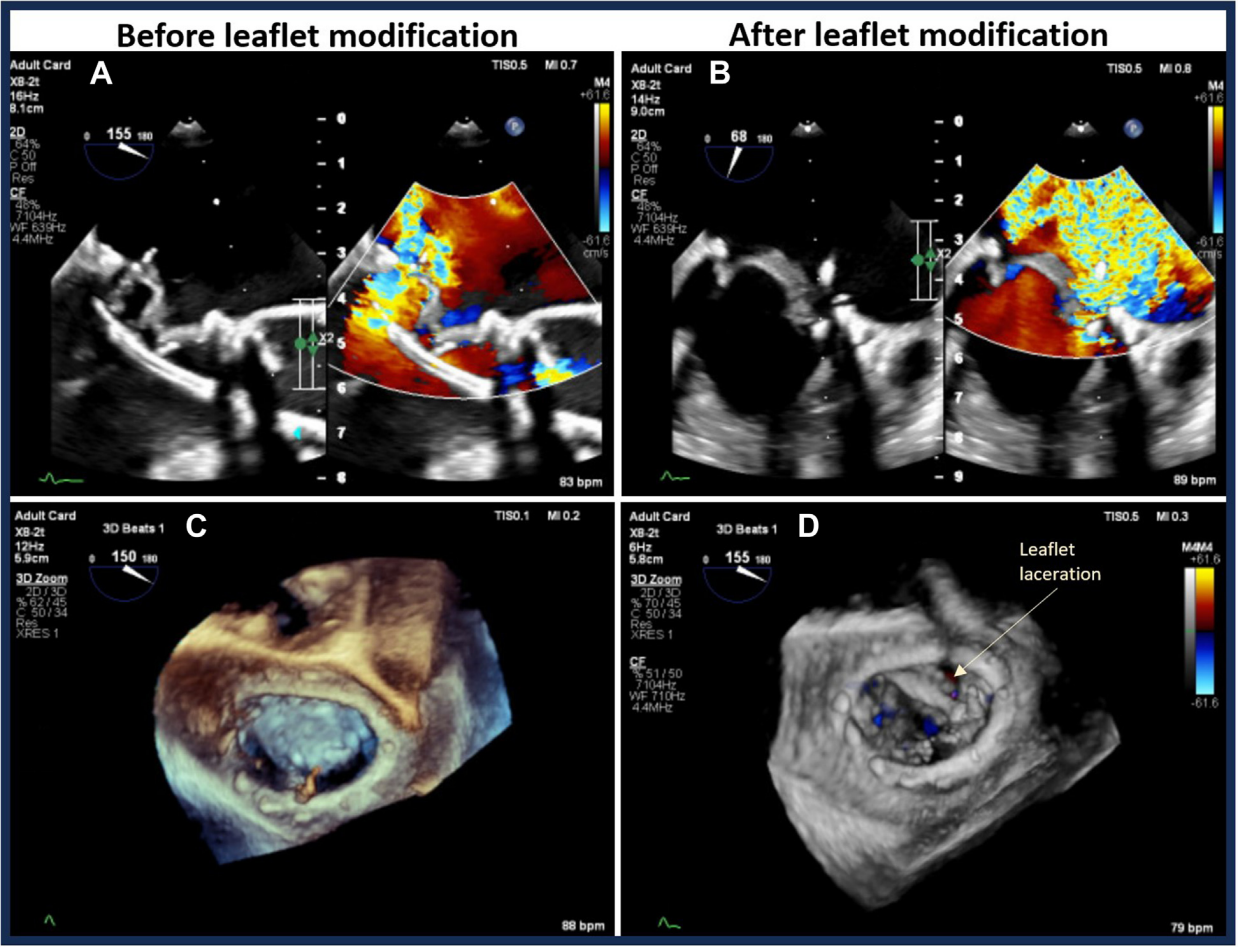
Anterior Mitral Valve Leaflet Laceration Before (A and C) and after (B and D) modification. (A) The “flying V” configuration of catheters and wire. (B) The impact of leaflet laceration with significant worsening of mitral regurgitation on color Doppler. (C and D) The appearances on 3-dimensional transesophageal echocardiography before (C) and after (D) leaflet laceration.

**Figure 4:**
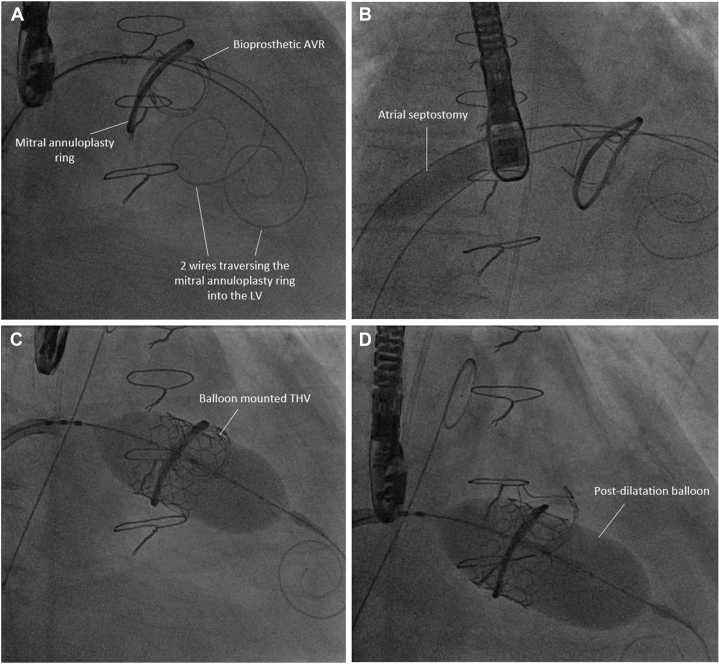
Fluoroscopic Images of the Implantation of the Transcatheter Heart Valve The mitral annuloplasty ring is traversed by 2 wires (A), and atrial septostomy is performed with a 14-mm balloon (B). The balloon-expanding THV was deployed under rapid burst pacing (C) and post-dilatated with 4 cc overfill (D). LV = left ventricle.

**Figure 5:**
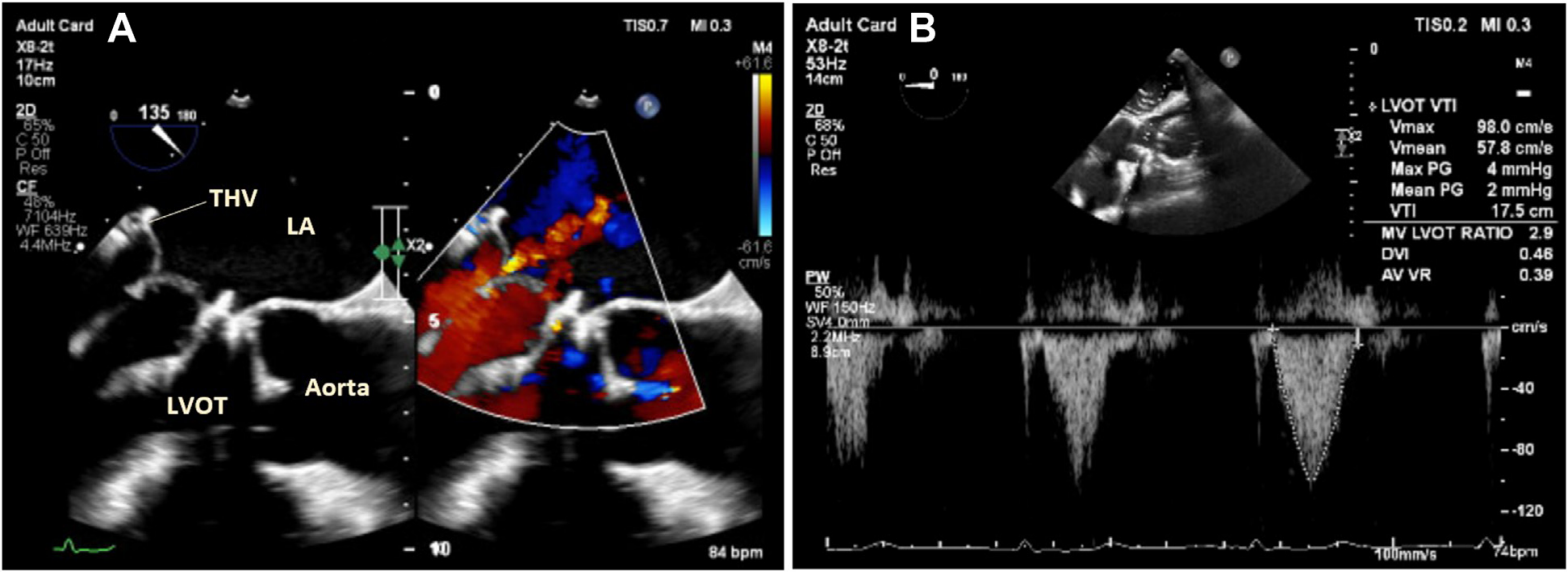
2-Dimensional Transesophageal Echocardiography The positioning of the transcatheter heart valve (THV) in relation to the left ventricular outflow tract (LVOT) with minimal transvalvular regurgitation (A) and a continuous-wave Doppler through the LVOT demonstrating no significant gradient (B). LA = left atrium.

**Figure 6:**
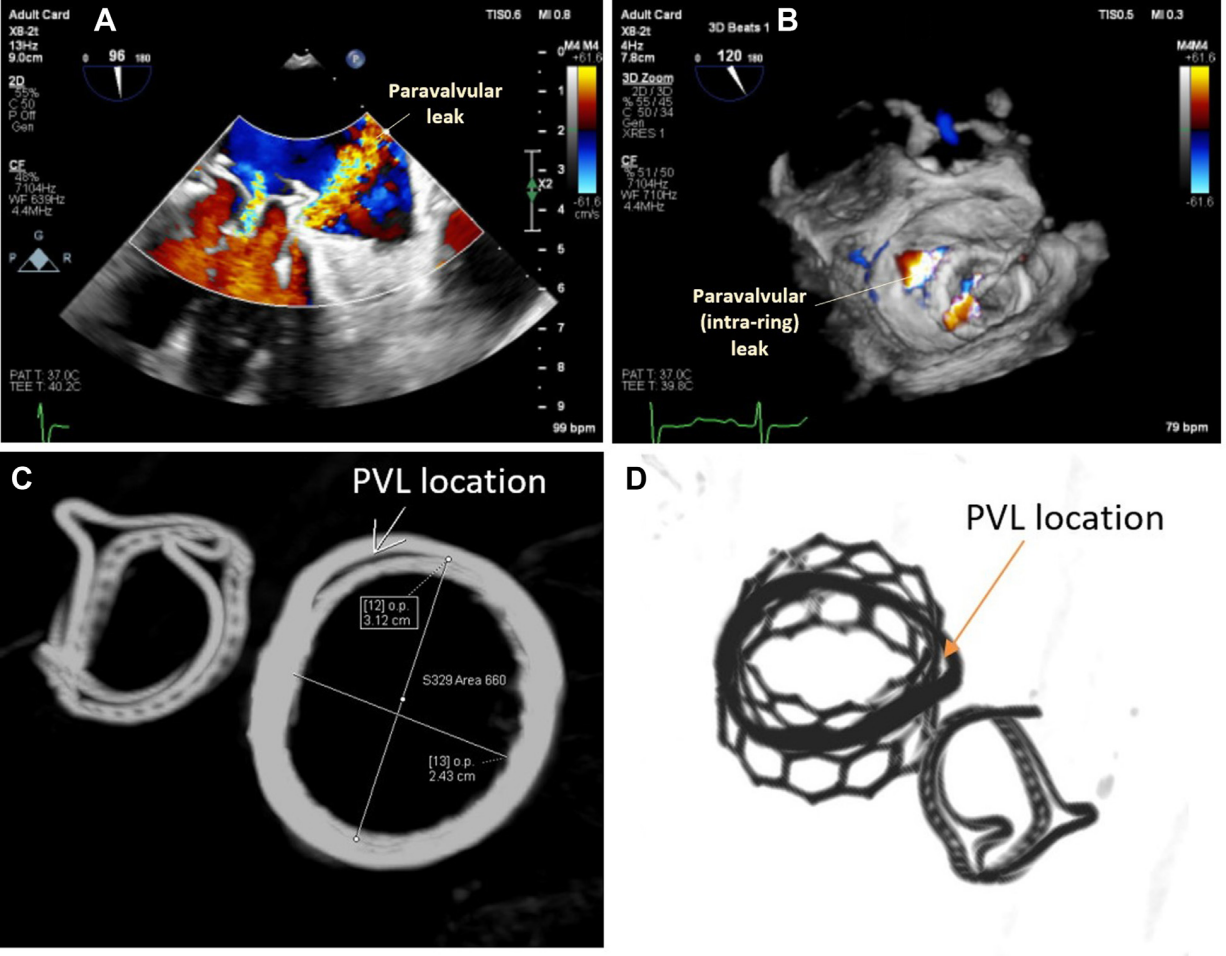
Paravalvular (Intra-Ring) Leak Between the THV and the Annuloplasty Ring (A and B) Transesophageal echocardiography and (C and D) computed tomography. PVL = paravalvular leak.

**Figure 7:**
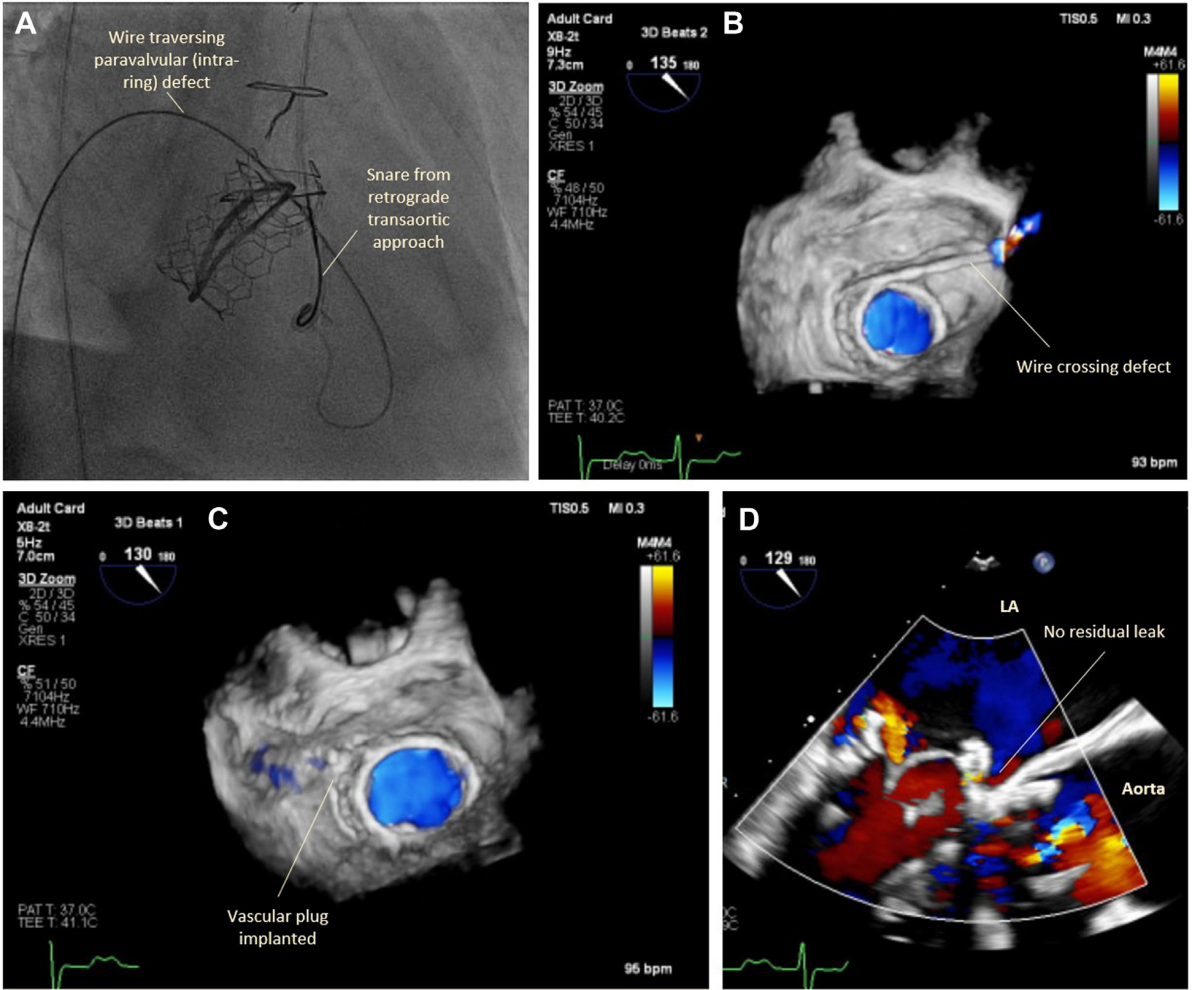
Closure of the Paravalvular Leak Closure of the paravalvular leak by (A and B) traversing the defect with a wire and (C) implantation of a vascular plug. (D) The final result on transesophageal echocardiography with color Doppler is shown, demonstrating no significant leak. LA = left atrium.

**Figure 8:**
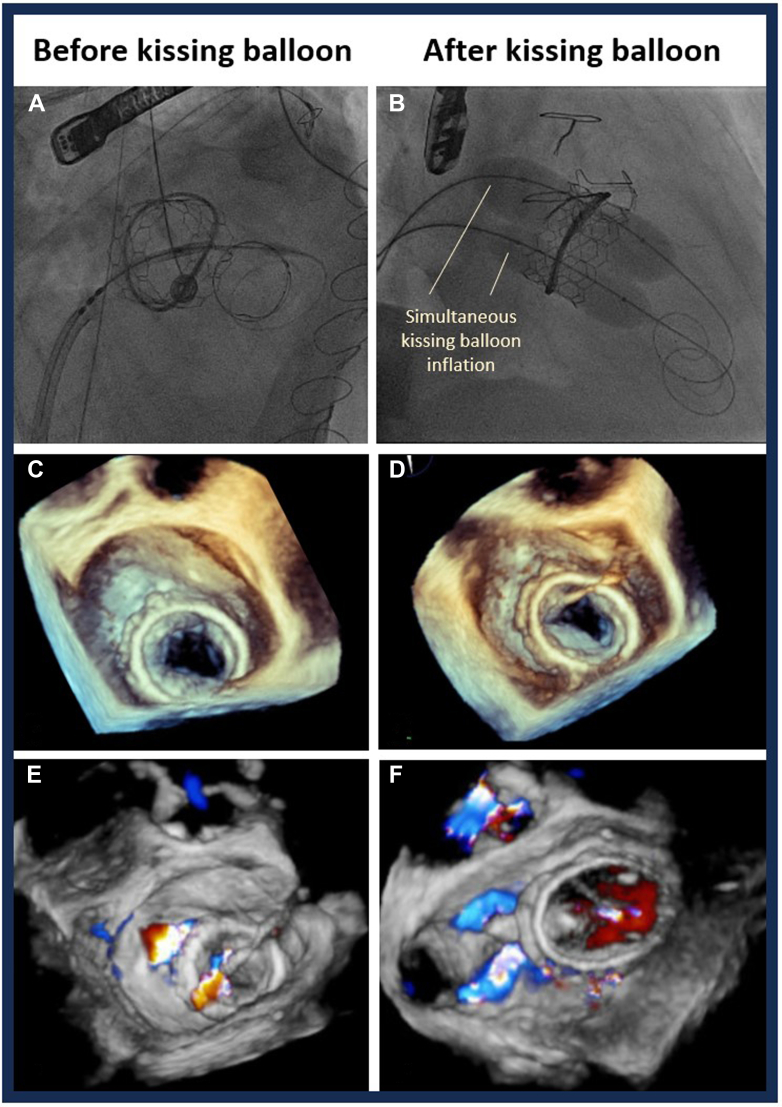
The Effect of Kissing Balloon Dilatation on THV Appearance Appearance of the transcatheter heart valve before (A, C, E) and after (B, D, F) kissing balloon dilatation in the angiographic (A and B) and 3-dimensional transesophageal echocardiography views (C to F) with (E and F) and without (C and D) color Doppler. (D and F) A significantly more elliptical shape compared with the more circularized appearance seen in C and E.

The procedure was performed electively with the patient under general anesthesia. TEE-guided transseptal puncture was performed with radiofrequency ablation. A balloon-tipped catheter was floated across the mitral valve and into the aorta. An Astato wire (Asahi Intecc Medical) was passed through the catheter, snared, and externalized via a left femoral artery sheath. A “flying V” configuration was formed using the Astato wire insulated by 2 guiding catheters. The sheath tips were positioned in the center of the mitral valve orifice to act as a pivot for centerline laceration. The catheters were then withdrawn to electrosurgically lacerate the AMVL from tip to base with the annuloplasty ring as the backstop to prevent injury to the aortomitral junction (Figure 2). Successful laceration of the AMVL was confirmed on TEE (Figure 3). After removal of the catheters and externalized wire, the mitral valve was re-crossed with a Safari wire and septotomy was performed with a 14-mm balloon. The Baylis sheath was then replaced with a 16-F E-sheath, following which a reverse-mounted 29-mm Sapien S3 was deployed (under rapid burst pacing) using a 30/70 distribution to reduce the risk of LVOT obstruction, 10 cc overfilled, and post-dilated (Figure 4). An excellent position was achieved with no LVOT obstruction or significant central MR (Figure 5). The patient was discharged, but he was readmitted with significant hemolytic anemia (Hb 62 g/dL, bilirubin 86 μmol/L). Repeated CT and TEE found moderate MR due to an intra-ring paravalvular jet (Figure 6). There was no central MR. Consequently, after more discussion with the heart team, a further procedure was performed to address the residual MR. Transfemoral transseptal crossing was performed, the leak between the annuloplasty ring and THV was traversed, and an 8-mm Amplatzer vascular plug 4 (Abbott Cardiovascular) was deployed (Figure 7). The THV was then crossed with 2 Safari wires and kissing balloon dilatation was performed (20 mm and 18 mm Cristal) to modify the circular THV into the elliptical shape of the annuloplasty ring, without forcing circularization of the annuloplasty ring, producing an excellent final result with only trivial residual MR (Figure 8).

## Outcome

The patient was transferred to the intensive care unit for recovery. There was no immediate postprocedural complication, and the patient remained hemodynamically stable.

## Discussion

This is, to our knowledge, the first described use of the kissing balloon dilatation approach to deform the THV to better fit the annuloplasty ring. In this case, an extra-large 36-mm annuloplasty ring was implanted. Thus, despite deploying the largest available 29-mm Sapien S3 valve with overfilling, and rigorous conventional post-dilatation, the circular Sapien S3 valve left a clinically significant paravalvular jet causing symptomatic hemolytic anemia. Although several factors should be considered, including skirt leak from atrial implantation and iatrogenic ring dehiscence, we felt that the CT and echocardiography demonstrated that the most likely cause was malapposition of the valve within the ring. Kissing balloon dilatation was performed after the implantation of a vascular plug between the annuloplasty ring, and therefore it is unclear to what extent the reduction of the PVL was due to the former or the latter. However, the result shown in this case highlights that the 2 techniques can be successfully performed concomitantly with echocardiographic and CT evidence of better apposition of the THV to the annuloplasty ring. Furthermore, kissing balloon dilatation of the THV may also improve the stability and sealing of the plug within the defect. While kissing balloons have been used in other structural interventions (eg, to prevent neo-LVOT obstruction in TMVR,^8^ to perform mitral valvuloplasty,^9^ or lithotripsy to facilitate TAVR^10^), this is, to our knowledge, the first reported use to improve conformity of the circular valve within a D-shaped annuloplasty ring to avoid inadvertent circularization of the annuloplasty ring which could potentially cause ring dehiscence. Although we demonstrate that PVL may be reduced by this technique, there is a risk of overdilatation or distortion which could introduce a central leak. For this reason, we recommend selecting appropriately sized kissing balloons. Furthermore, overdilatation with a single large balloon (in contrast to 2 kissing balloons) may distort and circularize the ring which would reduce the risk of intra-ring regurgitation but increase the risk of a para-ring leak. Further studies are required to validate the efficacy of the kissing balloon technique to reduce intra-ring regurgitation after TMVR within large annuloplasty rings.

**Visual Summary:**
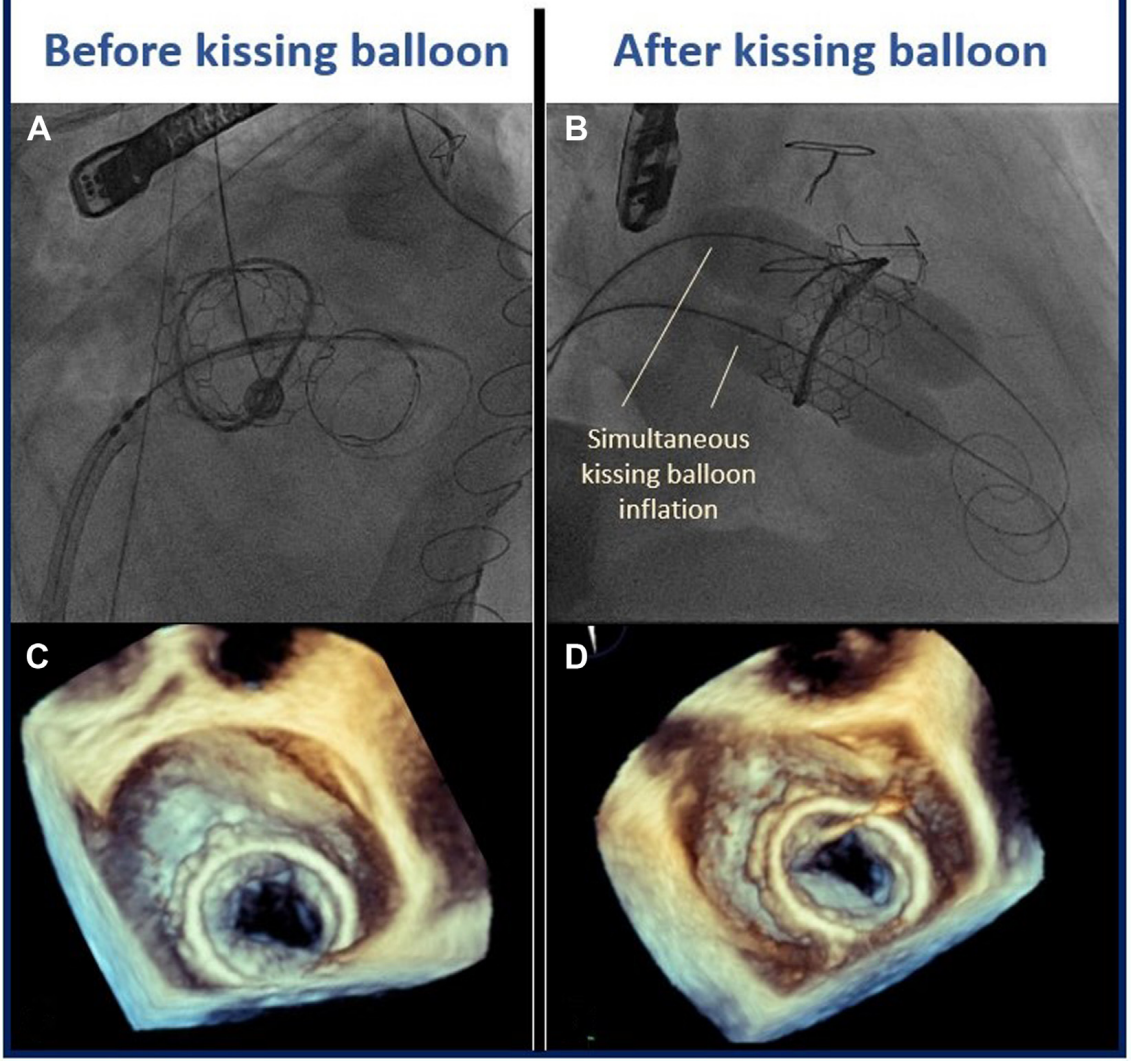
Kissing Balloon Inflation to Improve Conformity of Valve Within the Annuloplasty Ring

## Funding Support and Author Disclosures

Dr Doshi is a proctor for Edwards Lifesciences. All other authors have reported that they have no relationships relevant to the contents of this paper to disclose.
